# 

**Published:** 1871-04

**Authors:** 


					﻿PUBLISHED MONTHLY, AT $3 00 PER AI^^NiJiM IN ADVANCE.
-	■	• I .
4	"T yv TV .-V	‘
0)
ci	*	"0
i	■ ' -■	s
= — ;!
O	----- .	_____ ...
I Medical and Surgical Journal. |
M-	Xi
--
a>
r+
•-	®
Q>	-	a.
rt	o
•	o
,	o
.■^	'3
5	EDITED BY
?•
£ •
r	W. F. WESTMORELAND, M. D.,
rt	.	•
>, Prof, of fhe Prinripl<>.s ninl Proctiee of Siirijery iii th^ Afinnto ifeillcol ('oUrye.
t/>	-
c	C-
a,	AND	>■«:
’	I®?
O	I <■.
s'	J. G. WESTMORELAND, M. D.,
£ Prof, of .Maierift APtlim (nul Tker<.ipe,utics in Aflanto. .yiediral Colleijp. 3
S	•	8
5	■	. '■•
o	-	(0
. _________________ □
y,-	r+
S	• Pax et Scientia, sed veritas sine timore.	®
«_
rt	O
42	C
c
®	-Z.
>
r2	*“
________________	_____________________________________ ' in'
A^ol.TXB.______APRIL. 1871.	■	No. l. l
~	X- ..	(A
O --------:--	---------- --- , -IJI- ,	, ■ -- ■ ■ -. -■— - __ ■ -	• G ,
o “ “	.	Q.
I	6^/•?
!=■	■	.	'2.
Z5	■	-5 .
L	oi
LxJ
a	0
ATLANTA, GEORGIA;	«
' Q	the TEVE GEOEGIAN book and .TOB printing ESTABl.IsmiEN'r.	•	:
0-	1871.
EACH NUMBER CONTAINS SIXTY-FOUR PACES-
				

